# Levels of Inflammatory and Bone Metabolic Markers in the Gingival Crevicular Fluid of Individuals Undergoing Fixed Orthodontic Treatment in Comparison to Those Utilizing Invisalign

**DOI:** 10.3390/medicina59122107

**Published:** 2023-12-01

**Authors:** Abdullah A. Alnazeh, Muhammad Abdullah Kamran, Yahya Aseeri, Mohammad Raji Alrwuili, Mohammed Ahmed Aljabab, Eisha Abrar Baig, Mohammad Shahul Hameed

**Affiliations:** 1 Department of Pedodontics and Orthodontic Sciences, College of Dentistry, King Khalid University, Abha 62529, Saudi Arabia; 2 Specialist in Restorative Dentistry (AEGD), Ministry of Health, Abha 7588, Saudi Arabia; 3 Orthodontic Department, Qurayyat Specialized Dental Center, Al-Qurayyat 77453, Saudi Arabia; 4 Dow International Dental College, Dow University of Health Science, Karachi 74200, Pakistan; 5 Department of Diagnostic Sciences and Oral Biology, College of Dentistry, King Khalid University, Abha 62529, Saudi Arabia

**Keywords:** invisalign, fixed orthodontic therapy, bone metabolism biomarker, gingival crevicular fluid

## Abstract

*Background and Objectives*: Evaluation of the levels of cytokine and bone metabolic biomarkers (BMBs) in patients receiving fixed orthodontic therapy (FOT) and Invisalign. *Materials and Methods*: Sixty participants were enrolled after meeting the predefined inclusion criteria. Patients then underwent either FOT or Invisalign by allocating them randomly to each group (n = 30). The basic periodontal assessment was performed, including the plaque index (PI), gingival index (GI), and bleeding on probing (BoP), at baseline and again after 4 weeks. Gingival crevicular fluid (GCF) samples were taken from each individual at baseline and after 4 weeks. An enzyme-linked immunosorbent assay (ELISA) technique was used to determine the cytokine and BMB levels. An unpaired *t*-test compared the FOT and Invisalign group’s means and SDs. Paired *t*-tests examined the difference between T0 baseline and T1. *Results*: Patients treated with either FOT or Invisalign presented no statistically significant difference in terms of periodontal parameters such as PI, GI, and BoP (*p* > 0.05). The levels of IL-6 were significantly higher in patients treated with FOT as compared to Invisalign at T1 (*p* < 0.05) The other tested cytokines, IL-10, 13, 17, and GM-CSF, were not significantly different in either the FOT or Invisalign group at baseline and 4 weeks follow-up (*p* > 0.05). Regarding BMBs, it was detected that NTx and OC levels in both of the investigated groups were not significantly different at baseline and after 4 weeks (*p* > 0.05). However, NTx levels rose significantly (*p* < 0.05) and OC levels fell from T0 to T1. *Conclusions*: FOT and Invisalign displayed comparable outcomes in terms of cytokine and BMB levels. However, only IL-6 and NTx were significantly different at week 4 from baseline.

## 1. Introduction

The process of orthodontic tooth movement (OTM) is predominantly governed by a biological mechanism involving bone resorption and apposition. This intricate process is set in motion by the mechanical stresses applied through orthodontic appliances [1]. These mechanical forces act as initiators, prompting a cascade of biological responses within the surrounding tissues. Bone resorption facilitates movement of the tooth and creates space, while bone apposition contributes to the establishment of new bone in response to the altered mechanical environment. Together, these coordinated biological processes underlie the dynamic nature of orthodontic tooth movement [2]. The clinical effectiveness of OTM is intricately tied to the differentiation of osteoclasts and the activation of cytokines. This collaborative interaction between osteoclasts and cytokines plays a pivotal role in orchestrating the bone remodeling mechanism [3,4]. Osteoclasts, which are specialized cells responsible for bone resorption, are crucial for creating a conducive environment for tooth movement [5]. Simultaneously, cytokines, which are signaling proteins involved in immune and inflammatory responses, contribute to the regulation of this process by mediating communication between cells. The harmonious interplay of osteoclast differentiation and cytokine activation is fundamental to the success of bone remodeling, ultimately ensuring effective and controlled orthodontic tooth movement [6,7].

Gingival crevicular fluid (GCF), an inflammatory exudate, was first described by Alfano [8]. The constituents of GCF include proteins, cytokines, bacterial antigens, electrolytes, tiny chemical compounds, and enzymes derived from both the host and bacterial origins. Cytokines and bone metabolic biomarkers (BMBs) are essential factors in the process of attaining tooth mobility in the context of orthodontic therapy [9,10]. Cytokines, within the context of biological responses, constitute a diverse group of low-molecular-weight proteins. These proteins are secreted in an autocrine or paracrine manner, responding to localized force or stress within the physiological environment. Their role extends to regulating various cellular activities and orchestrating complex signaling pathways, thereby contributing to the dynamic processes that occur in response to external stimuli [11]. Furthermore, molecular markers of bone metabolism serve as sophisticated tools for gauging the intricate dynamics of bone remodeling. These markers provide valuable insights into the molecular events associated with the turnover of bone tissue, shedding light on the balance between bone resorption and formation. Utilizing these markers enhances our understanding of the underlying mechanisms governing orthodontic tooth movement and bone responses during therapeutic interventions [12].

The increasing emphasis on patients’ cosmetic preferences has driven the development of Invisalign, a clear aligner therapy option [13]. Compared to fixed orthodontic therapy (FOT), Invisalign stands out by providing improved oral hygiene maintenance, which enhances both aesthetics and comfort for patients [14]. Aligners deliver periodic orthodontic stresses that modify the conventional phases of tooth movement as previously elucidated by Krishnan and Davidovitch [15]. While Invisalign has limitations in addressing specific malocclusions and can be associated with higher costs, it continues to capture patients’ attention. Some authors argue that periodic orthodontic stresses caused by malocclusions result in the lower activation of cytokines and bone matrix turnover levels compared to fixed orthodontic therapy (FOT) [16,17]

At present, evidence is scarce about the activation of various cytokines and BMBs in individuals enduring treatment with labial fixed appliances and Invisalign. Therefore, it was predicted that there would be no significant difference in the cytokine levels: IL-6, 10, 13, 17 and GM-CSF (granulocyte-macrophage colony-stimulating factor); and BMBs: NTx (N-telopeptides) and OC (osteocalcin), among the individuals subjected to FOT and Invisalign. Furthermore, it was also postulated that there would be no significant difference in cytokines and BMBs in both the groups from T0 to T1. Hence, the purpose of the existing work is to investigate the level of inflammatory mediator cytokines and BMBs in participants receiving orthodontic care using FOT or Invisalign.

## 2. Material and Methods

### 2.1. Study Design and Participants

The study enrolled participants from the orthodontics department of the University of Health Sciences (UHS) who were receiving orthodontic treatment with FOT or Invisalign. The ethical committee approved the study under IRB#FC/0039-117, 18 February 2023. The research adhered to the principles outlined in the Declaration of Helsinki [18]. Before commencing the study, participants were required to give their consent, which was obtained through both written and verbal means.

### 2.2. Sample Size and Allocation of the Patients

For the calculation of the sample size, it was initially deemed that 20 participants would suffice for each group, considering a significance level of 0.05 and a study power of 0.80 to detect a significant difference. However, to account for the potential underestimation of power, anticipated sample loss, and possible dropouts during follow-up, the determined sample size was adjusted to 30 participants for each group, resulting in a total of n = 60 participants. A group of sixty individuals aged between 18 to 32 years old (mean age 25 ± 3) was selected for the study. Among these sixty participants, thirty were assigned FOT with a fixed labial appliance (comprising 18 females and 12 males), while the remaining thirty individuals were assigned to Invisalign treatment (including 20 females and 10 males).

### 2.3. Inclusion Criteria

The selection criteria for participants in the study were as follows. Health status: prospective participants should be free of systemic illnesses and should not have a smoking habit. Medication history: there should be no documented use of nonsteroidal anti-inflammatory drugs (NSAIDs) or antibiotics in the six months preceding their inclusion in the study. The study exclusively enrolled patients diagnosed with crowding within the range of 2.1 to 4.0 mm to ensure a consistent level of malocclusion among participants. Oral health assessment: participants should have a gingival index (G1) score of at least <1, indicating good gingival health, and a generalized pocket depth of ≤3, signifying healthy gums. Radiographic evaluation: additionally, digital radiographs were examined, and no signs of crestal bone loss were observed.

### 2.4. Exclusion Criteria

Individuals with conditions such as kidney failure, HIV, liver disease, periodontal disease, and inadequate dental hygiene were not included in this study. Before the research commenced, all participants received education on oral hygiene practices, including guidance on brushing techniques, the use of fluoridated water and toothpaste, and the recommended brushing frequency. Two weeks before the research began, prophylactic supra and subgingival scaling were carried out as preparatory measures. Regular follow-up sessions were conducted to provide ongoing support and encouragement to the participants throughout the trial.

### 2.5. Periodontal Parameter Monitoring

The baseline periodontal assessment involved recording the plaque index (PI), gingival index (GI), and bleeding on probing (BoP) scores for each patient. To assess the PI in both the upper and lower arches, specific reference teeth were selected. These included the first molar and lateral incisor in the right upper quadrant, as well as the first bicuspid in the left quadrant. For the evaluation of the GI, each tooth was assigned scores based on four specific aspects: the labial, lingual, distal, and mesiolingual sides. The same assessments were repeated after 4 weeks to track any changes or improvements.

### 2.6. Measuring and Collecting GCF

Gingival crevicular fluid (GCF) samples were collected at two time points: baseline before therapy (T0) and after 4 weeks (T1), as indirect resorption typically begins around the 28-day mark. To ensure consistency in the sample collection, the site chosen for GCF collection was where gingival irritation was minimal and uniform among all participants, specifically proximal to the canines in the upper arch. To prevent contamination, sterile gauze was used to ensure isolation. A 1 µL volume of GCF was gently obtained by carefully placing a pipette in the sulcus, making slight contact with the gingival border, using an aseptic technique. Any pipette that came into contact with blood or saliva during the procedure was excluded from the analysis. Subsequently, the collected GCF was transferred to 0.5 mL Eppendorf tubes, centrifuged at 3000 rpm, and stored at −80 °C for 10 min before conducting the test. Throughout the entire process, strict blinding procedures were maintained to ensure the accuracy and integrity of the data.

### 2.7. Analysis of Cytokines Using ELISA (Enzyme-Linked Immunosorbent Assay)

The GCF obtained from all the patients who underwent FOT and Invisalign therapy were subjected to thawing at room temperature for ELISA. The BMBs, i.e., N-terminal telopeptide (NTx) and osteocalcin (OC), together with the inflammatory cytokines (Interleukin (IL)—6, 10, 13, 17 and GM-CSF) were evaluated using the ELISA technique, following the guidelines provided by the manufacturer. Samples and standards were mixed in matched wells followed by incubation overnight at 4 °C. Each well received 0.1 mL of 1% biotinylated anti-human CRP detector antibodies. The incubation was performed at a room temperature of 25 °C for 60 min in the dark. Before incubating, 0.1 mL of 1% HRP–Streptavidin solution was incorporated into each well for 45 min at 25 °C. The sensitivity of ELISA for all GCF cytokines was above 99.1%.

### 2.8. Statistical Analysis

Statistical analysis was performed using SPSS v.18 (IBM, Chicago, IL, USA). The Kolmogorov–Smirnov test determined data normality. The cytokine levels were presented in terms of means and standard deviations (SDs). We used paired sample *t*-tests to compare the FOT and Invisalign means and SDs. We also used paired *t*-tests to examine the difference between the T0 baseline and T1, keeping the significance level at *p* = 0.05.

## 3. Results

**Figure 1:**
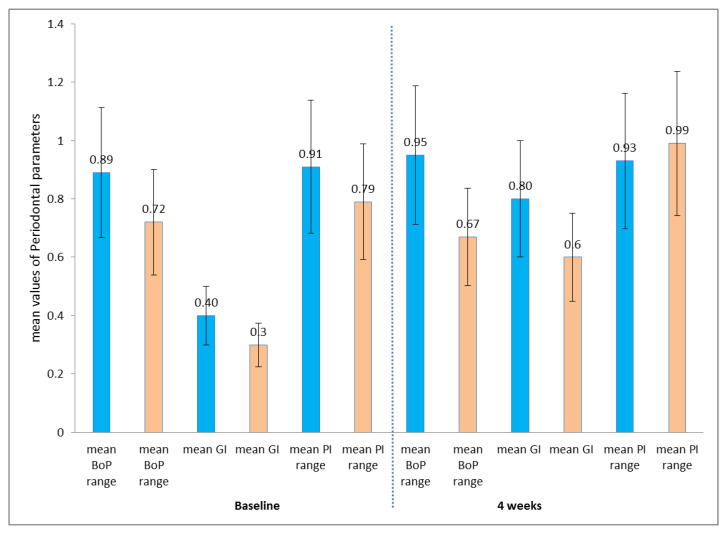
Demonstrates initial periodontal measurements and those taken after 4 weeks for individuals undergoing FOT (fixed orthodontic treatment) and those using Invisalign. BoP: bleeding on probing; GI: gingival index; PI: periodontal index.

Table 1 and Figure 1 display the periodontal parameters at baseline and after 4 weeks in participants undergoing orthodontic treatment using a fixed labial appliance and Invisalign. Results indicate that patients treated with FOT or Invisalign presented no statistically significant difference in terms of periodontal parameters such as PI, GI, and BoP (*p* > 0.05).

**Figure 2:**
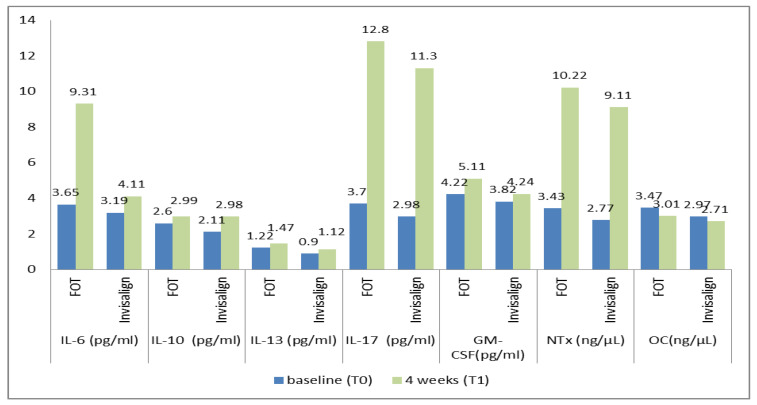
Cytokine and bone metabolism biomarker levels in the gingival crevicular fluid (GCF) of 30 patients each undergoing FOT (fixed orthodontic treatment) or Invisalign. The measurements are taken at two time points: baseline (T0) and 4 weeks later (T1), and statistical analysis is performed using both u—paired and paired *t*-tests. GM-CSF (Granulocyte-macrophage colony-stimulating factor); NTx (N-telopeptides); OC (osteocalcin); FOT (fixed orthodontic therapy); IL (interleukin).

Table 2 and Figure 2 present the level of different cytokines and BMBs in the FOT and Invisalign groups at T0 and T1. It was observed that patients treated with FOT had significantly higher levels of IL-6 as compared to the Invisalign group at T1 (*p* < 0.05). However, its level at T0 was comparable among both groups. Furthermore, it was also established that other cytokine mediators, IL-10, 13, 17, and GM-CSF, were not significantly different in either the FOT or Invisalign group at baseline and at 4 weeks follow-up (*p* > 0.05). Regarding BMBs, it was observed that NTx and OC levels in both the investigated groups were not significantly different at baseline (*p* > 0.05). However, NTx level rose significantly (*p* < 0.05) and OC levels decreased from T0 to T1.

## 4. Discussion

The present study was based on the hypothesis that there would be no significant difference in the cytokine levels (IL-6, 10, 13, 17, and GM-CSF) and BMBs (NTx and OC) among the individuals subjected to either FOT or Invisalign. Furthermore, it was also postulated that there would be no significant difference in cytokine levels and BMBs in both the groups from T0 to T1. The primary suggested hypothesis was partially accepted as levels of IL-6 were found to be higher in the FOT group as compared to the Invisalign group at the week 4 follow-up. However, the second assumption was partially rejected as IL-6, IL-17, IL-10 NTx, and OC levels changed from baseline to week 4. The rest of the other mediators displayed no temporal relation from T0 to T1. The assessment of the extent of remodeling in periodontal tissues during orthodontic treatment by measuring the concentrations of biochemical mediators could potentially serve as a valuable clinical procedure due to the significant roles of these mediators in the process of tooth movement [11,19].

The mechanism of tooth movement is a multifaceted process involving the activation of various cell subpopulations and soluble inflammatory chemicals. These elements work in harmony to trigger bone resorption [19,20,21]. In the context of inflammatory cytokines analyzed in both the control and experimental groups, it was observed that there were no statistically significant alterations in the levels of most of the mediators, which include IL-10, IL-13, IL-17, and GM-CSF, except for IL-6. Regarding the changes in the levels of these mediators over 4 weeks, it was noted that there was no temporal fluctuation in the levels of GM-CSF, IL-10, and IL-12. However, there was an increase in the levels of IL-6 and IL-17 observed at T1. These findings align with the results of previous research conducted by Kamran and colleagues [12]. According to their findings, IL-6 levels were found to be higher in patients undergoing fixed orthodontic therapy (FOT) compared to those treated with clear aligners. Additionally, Başaran et al. noted that the expression of the IL-6 protein was influenced by both the duration and magnitude of the treatment, indicating a time-dependent and magnitude-dependent relationship [22]. Previous research has suggested that IL-17 can exhibit synergistic effects when it interacts with other cytokines, resulting in the increased production of IL-6. IL-17 belongs to a newly identified group of cytokines that play a critical role in bone remodeling and the regulation of cell-mediated immune responses [23,24]. Results of the latest research conducted by Allgayer et al. and Chami et al. are in agreement with the outcomes of the existing work [14,25]. However, data related to the impact of Invisalign on IL-17 levels are scarce, and therefore need further investigation.

It was found that the levels of GM-CSF were similar in both groups at T0 and T1. However, despite its importance as a pro-inflammatory cytokine in bone remodeling, the exact role of GM-CSF remains unclear from the previous academic literature. Additionally, IL-10 and IL-12, which are known as anti-inflammatory mediators for bone resorption, showed no differences between the groups. Furthermore, there were no temporal variations noted in the levels of IL-10 and IL-12 after 4 weeks of treatment. Prior studies by Nunes and colleagues and Gastel and coworkers revealed no significant difference in the levels of IL-10 and IL-13 over 4 weeks after conventional orthodontic therapy [26,27]. Another recent work by Almeida et al. showed small increases in IL-10 concentration levels on the third day of orthodontic activation, followed by a drop without statistically significant changes [28].

Concerning BMBs, it was reported that levels of NTx at both baseline and T1 demonstrated comparability between the control and test groups in the study. Despite this initial similarity, a noticeable temporal increase in NTx levels was observed in both groups within the 4-week timeframe. This observed phenomenon of NTx resorption is attributed to the distinctive amino acid sequences and organizational characteristics of the cross-linked alpha-2 N-telopeptide [29]. This finding is consistent with the outcomes of a study conducted by Alfaqeeh et al., further supporting the notion that NTx levels undergo a temporal increase, possibly reflecting the dynamic and responsive nature of bone remodeling processes in response to various stimuli or interventions. An understanding of NTx dynamics is integral in assessing bone metabolism and the impact of orthodontic treatments on bone resorption markers [30]. OC is a noncollagenous matrix protein synthesized by osteoblasts, and it is well-recognized for its substantial influence on both bone resorption and mineralization processes. Recent research conducted by Griffiths and colleagues revealed no significant fluctuations in OC levels over 4 weeks [31]. However, it is important to note that data concerning the effects of Invisalign on bone markers remains insufficient and requires further investigation to better understand its impact.

In the context of this study, it is important to acknowledge its limitations. The assessment of tooth movement using both FOA and aligners has the potential to establish a more conclusive link with the response of inflammatory biomarkers. It is widely understood that individuals respond differently to mechanical loading, influenced by variables such as age, gender, and bone density, which can contribute to varying outcomes. Moreover, the short duration of the study represents a significant constraint. To advance our understanding, it is recommended to undertake further clinical trials, potentially incorporating a split-mouth design, to enhance the generalizability and applicability of the findings from this present study.

## 5. Conclusions

The results of the study indicate that both fixed orthodontic therapy and Invisalign treatment yielded comparable results in terms of cytokine and bone metabolic biomarker levels. However, there was a notable rise in IL-6 and NTx levels at week 4 in comparison to the baseline in both cohorts. Further analysis and interpretation are required to fully comprehend the possible influence of these findings on treatment outcomes and the well-being of patients, highlighting their clinical significance.

## Figures and Tables

**Table 1:** Periodontal parameters at baseline and 4 weeks in participants undergoing FOT and Invisalign.

Periodontal Parameters	Baseline N = 30 Each		4 Weeks N = 30 Each		*p*-Value
	FOT	Invisalign	FOT	Invisalign	
**Mean BoP range**	0.89 (0.3–1.2)	0.72(0.3–0.9)	0.95 (0.4–1.1)	0.67 (0.2–0.6)	**0.312**
**Mean GI**	0.4 (0.2–1.0)	0.3 (0.3–0.8)	0.8 (0.3–1.0)	0.6 (0.2–0.4)	**0.574**
**Mean PI range**	0.91 (0.4–1.3)	0.79 (0.52–1.3)	0.93 (0.6–1.4)	0.99 (0.4–1.1)	**0.215**

FOT: Fixed orthodontic therapy; BoP: bleeding on probing; GI: gingival index; PI: periodontal index.

**Table 2:** Levels of cytokines and bone metabolism biomarkers in GCF (pg/mL) of patients undergoing FOT and Invisalign (n = 30 each) at baseline T0 and after 4 weeks T1 using un-paired and paired *t*-tests.

Biomarkers in GCF	Groups	Mean ± SD Baseline (T0)	Mean ± SD 4 Weeks (T1)	*p*-Value
IL-6 (pg/mL)	FOT	3.65 ± 1.21	9.31 ± 1.47 *	0.014
	Invisalign	3.19 ± 0.42	4.11 ± 0.33 *	0.024
IL-10 (pg/mL)	FOT	2.6 ± 0.42	2.99 ± 0.61	0.028
	Invisalign	2.11 ± 0.21	2.98 ± 1.01	0.025
IL-13 (pg/mL)	FOT	1.22 ± 0.11	1.47 ± 0.15	0.026
	Invisalign	0.9 ± 0.04	1.12 ± 0.11	0.025
IL-17 (pg/mL)	FOT	3.7 ± 1.23	12.8 ± 1.55	0.011
	Invisalign	2.98 ± 1.01	11.3 ± 1.23	0.017
GM-CSF (pg/mL)	FOT	4.22 ± 0.9	5.11 ± 1.41	0.024
	Invisalign	3.82 ± 0.7	4.24 ± 1.21	0.026
NTx (ng/µL)	FOT	3.43 ± 0.11	10.22 ± 0.15 *	0.017
	Invisalign	2.77 ± 0.8	9.11 ± 1.00 *	0.019
OC (ng/µL)	FOT	3.47 ± 0.34	3.01 ± 0.29	0.025
	Invisalign	2.97 ± 0.21	2.71 ± 0. 11	0.023

GM-CSF (Granulocyte-macrophage colony-stimulating factor); NTx (N-telopeptides); OC (osteocalcin); * FOT and Invisalign comparison paired sample *t*-test shows statistical significance.

## Data Availability

The data can be made available on request.
